# Traumatic brain injury: Mechanisms, manifestations, and visual sequelae

**DOI:** 10.3389/fnins.2023.1090672

**Published:** 2023-02-23

**Authors:** Steve H. Rauchman, Aarij Zubair, Benna Jacob, Danielle Rauchman, Aaron Pinkhasov, Dimitris G. Placantonakis, Allison B. Reiss

**Affiliations:** ^1^The Fresno Institute of Neuroscience, Fresno, CA, United States; ^2^NYU Long Island School of Medicine, Mineola, NY, United States; ^3^Department of Neuroscience, University of California, Santa Barbara, Santa Barbara, CA, United States; ^4^NYU Grossman School of Medicine, New York, NY, United States

**Keywords:** traumatic brain injury, light sensitivity, contrast sensitivity, neuroinflammation, visual acuity, headache, oxidative stress

## Abstract

Traumatic brain injury (TBI) results when external physical forces impact the head with sufficient intensity to cause damage to the brain. TBI can be mild, moderate, or severe and may have long-term consequences including visual difficulties, cognitive deficits, headache, pain, sleep disturbances, and post-traumatic epilepsy. Disruption of the normal functioning of the brain leads to a cascade of effects with molecular and anatomical changes, persistent neuronal hyperexcitation, neuroinflammation, and neuronal loss. Destructive processes that occur at the cellular and molecular level lead to inflammation, oxidative stress, calcium dysregulation, and apoptosis. Vascular damage, ischemia and loss of blood brain barrier integrity contribute to destruction of brain tissue. This review focuses on the cellular damage incited during TBI and the frequently life-altering lasting effects of this destruction on vision, cognition, balance, and sleep. The wide range of visual complaints associated with TBI are addressed and repair processes where there is potential for intervention and neuronal preservation are highlighted.

## 1. Introduction

**FIGURE 1:**
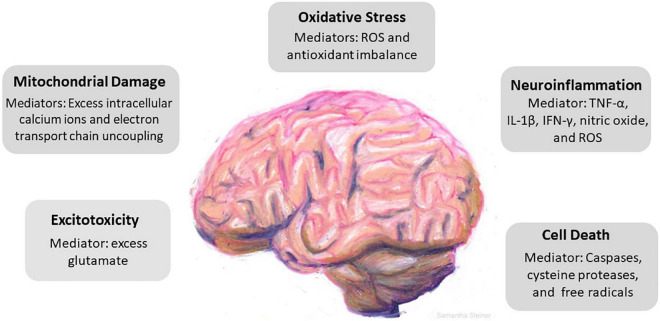
Cascade of cellular events driven by traumatic brain injury (TBI). TBI incites a series of responses that include excitotoxicity, mitochondrial damage, oxidative stress, neuroinflammation, and cell death. Key mediators in each pathological event is identified. Excess glutamate causes overactivation of N-Methyl-d-aspartate receptors (NMDAr) and α-amino-3-hydroxy-5-methyl-4-isoxazolepropionic acid receptors (AMPAr) which induces neuronal overexcitation and swelling. Mitochondrial damage follows as a result of excess influx of intracellular calcium and uncoupling of the electron transport chain. The precipitous rise of reactive oxygen species and the brain parenchyma’s relatively low antioxidant capacity promotes oxidative stress. Neuroinflammation induces secondary damage *via* the release of proinflammatory cytokines, chemokines, and inflammatory mediators and ultimately leads to cell death.

Traumatic brain injury (TBI) continues to be a major health concern in the United States and worldwide; often carrying long-term consequences that diminish quality of life and cause persistent cognitive impairment (Stocchetti and Zanier, 2016; Dewan et al., 2018). TBI can be mild, moderate or severe with about 80% of cases categorized as mild (Temkin et al., 2022). However, even mild TBI (mTBI) can lead to debilitating symptoms (McMahon et al., 2014; Ganti et al., 2019). It is misleading to assume that when the physical force applied to the head is weak, the consequences will be less. Some patients suffer substantially and for prolonged periods following mTBI, often with complaints of headache, dizziness, and memory issues (Prince and Bruhns, 2017; Hoffer and Balaban, 2019; Lu et al., 2020). Manifestations of TBI come on in phases, with a cascade of neurometabolic changes that affect the brain in complex and heterogeneous ways over a timespan ranging from days to years. Many TBI patients live with cognitive loss, behavioral problems, headaches and visual disturbances that interfere with their ability to work, socialize and fully participate in everyday life. This review will discuss our current knowledge of the functional and molecular deficits that result from TBI with an emphasis on the resulting impairments that affect the ability to carry out activities of daily living. Issues that link deficits in performance of key activities to visual sequelae involving focus, eyestrain, fatigue, and blurring will be highlighted. Beyond visual issues, cellular and molecular signaling pathways incited by TBI with resulting oxidative stress, inflammation, apoptosis, and autophagy are summarized in Figure 1 and discussed in detail below. Potential interventions to prevent or repair damage to the brain by limiting inflammation and facilitating neural regeneration are described.

## 2. Cellular response to traumatic brain injury

### 2.1. Excitotoxicity

A crucial feature of TBI is the acute phase, in which there is an immediate intractable excessive release of excitatory neurotransmitters that results from the stretching and tearing of brain tissue (Prins et al., 2013). This physical disruption leads to a cascade of pathological events called excitotoxicity (Hoffe and Holahan, 2022). The primary excitatory neurotransmitter released from presynaptic nerve terminals after an impact to cerebral tissues is glutamate (Chamoun et al., 2010; Thapa et al., 2021). In humans, up to a 50-fold increase in glutamate levels have been found, especially in patients with focal parenchymal contusions (Bullock et al., 1995; Bullock et al., 1998). The accumulation of glutamate in the synaptic cleft leads to repeated stimulation and overactivation of N-Methyl-d-aspartate receptors (NMDAr) and α-amino-3-hydroxy-5-methyl-4-isoxazolepropionic acid receptors (AMPAr) on the post-synaptic membrane (Lozano et al., 2015). With NMDAr activation, sodium channels also open and neurons swell (Wang et al., 2016). Glutamate shifts AMPAr toward a subtype that is more permeable to calcium ions (Spaethling et al., 2008). The opening of calcium channels and excess entry of intracellular calcium ions incites a destructive cascade. A multitude of catabolic enzymes are released, including phospholipases that destroy cell and mitochondrial membranes, proteases that interfere with cytoskeletal organization, and endonucleases that precipitate DNA fragmentation. This culminates in apoptosis and necrosis and cell death (Wang et al., 2016).

The presence of excessive glutamate post-TBI is primarily due to failure of glutamate re-uptake (Tasker, 2012; Baracaldo-Santamaría et al., 2022). Synaptic glutamate is removed by excitatory amino acid transporters (EAATs) that are expressed predominantly in astrocytes (Rothstein et al., 1996). Evidence has demonstrated a 40% decline in the expression of astrocytic sodium-dependent glutamate transporters GLAST (EAAT1) and GLT-1 (EAAT2) within 24 h following TBI, leading to a significant decrease in the resorption of glutamate (Rao et al., 1998; van Landeghem et al., 2006). Modulating effects of glutamate excitotoxicity has been explored as a mechanism for minimizing damage in TBI. However, pharmacologic antagonism of NMDA receptors has serious side effects including hallucinations, agitation, nausea (Morris et al., 1999; Ikonomidou and Turski, 2002).

Sowers et al. demonstrated excess glutamate oxidation in defined areas of injured hemispheres of rodents using matrix-assisted laser desorption ionization (MALDI)-imaging studies and metabolomics (Sowers et al., 2021). Glutamate is largely metabolized to glutamine in astrocytes. However, if oxidized, glutamate generates α-ketoglutarate, which yields ATP *via* the tricarboxylic acid (TCA) cycle. Sowers proposed that under conditions of great excess, glutamate oxidation may serve as a defense mechanism against glutamate excitotoxicity post- head injury and postulates that this mechanism may be leveraged to obtain better outcomes in TBI patients.

### 2.2. Mitochondrial damage

The massive accumulation of intracellular free calcium incited by depolarization promotes the activation of kinases and downstream enzymes capable of degrading phospholipids of the mitochondrial membrane (Jastroch et al., 2010). Although the mitochondria have protective calcium-buffering mechanisms in place, eventually the influx of calcium exceeds the buffering capacity and excess calcium ion accumulation in the mitochondria causes uncoupling of the electron transport chain located in the inner mitochondrial membrane (Duchen, 2012). This eliminates the concentration gradient between the mitochondrial matrix and the mitochondrial intermembrane space and reduces the capacity of the organelle to produce ATP (Bayir et al., 2005; McGovern and Barreto, 2021). The reduction of membrane potential *via* chemical uncoupling may serve as a defense against calcium overload by inhibiting uptake of calcium ions and serve a neuroprotective function in TBI, but loss of the gradient leads to permeabilization of the internal mitochondrial membrane as mitochondrial permeability transition pores open and ions and fluid flood the mitochondria. Eventually damage to the membrane becomes irreversible and bioenergetic collapse leads to cell death (Pandya et al., 2007).

The uncoupling of the electron transport chain also places oxidative stress on cells *via* free electrons that are easily trapped by oxygen. The subsequent buildup of reactive oxygen species (ROS) further intensifies the degradation of mitochondrial membrane phospholipids such as cardiolipin that are essential to maintain the selectivity and permeability of the inner mitochondrial membrane (Bayir et al., 2007; McGovern and Barreto, 2021). Furthermore, membrane degradation stimulates the cytosolic translocation of Cytochrome C, one of the mitochondria-dependent mechanisms for the activation of apoptosis or programmed cell death. Once in the cytosol, cytochrome C activates caspase proteins and other apoptotic proteins, eventually leading to apoptotic cell death (Kagan et al., 2005; Kumar, 2007; Chao et al., 2019).

### 2.3. Oxidative stress

Oxidative stress, defined as the imbalance of ROS and antioxidants, plays a crucial role in causing secondary damage post TBI (Birben et al., 2012). ROS levels in the brain rise precipitously following injury and may remain elevated for days afterward (Hall et al., 1993). Oxygen-derived free radicals known to place oxidative stress on the brain include hydrogen peroxide, superoxide anions, hydroxyl, and peroxyl radicals (Lewén et al., 2000; Hakiminia et al., 2022). These radicals can irreversibly oxidize macromolecules and injure cells. The brain parenchyma is especially vulnerable to oxidative damage due to its high oxidative metabolic activity, relatively low antioxidant capacity, and inadequate repair mechanisms (Shohami et al., 1997; Cernak et al., 2000; Ismail et al., 2020). In TBI, ROS are generated *via* mitochondrial leakage, the arachidonic acid cascade, and catecholamine oxidation (Hall et al., 1992; Marklund et al., 2001; Ismail et al., 2020). Neutrophils, which infiltrate the brain after TBI, are an important source of ROS and produce these molecules primarily by enzyme activity of nicotinamide adenine dinucleotide phosphate (NADPH) oxidase (NOX)2 (Liu et al., 2018; Kim et al., 2022). NOX are a family of multi-subunit transmembrane enzymes that reduce molecular oxygen into ROS and catalyze formation of the superoxide anion (Bedard and Krause, 2007). NOX2 is the primary producer of ROS in brain tissue and thus plays a crucial role in the development of secondary injury after TBI (Choi et al., 2012). Zhang et al. (2012) demonstrated NOX activation in microglia significantly contributed to neuronal damage within 24–48 h post TBI and further raised ROS production. Oxidative damage can manifest as lipid peroxidation of neuronal, glial, and vascular cell membranes as well as myelin (Zhang et al., 2012; Daradkeh et al., 2021). Consequently, NOX inhibition has become a therapeutic target of investigation as a means to reduce oxidative damage in TBI and has met with some success in murine models (Angeloni et al., 2015; Chandran et al., 2018; Wang et al., 2022). The severity of injury in TBI can be correlated with the degree of ROS-related tissue damage and mitochondrial dysfunction (Tavazzi et al., 2005; Lee et al., 2021).

Arachidonic acid, normally stored in phospholipid membranes within cells, is released from membranes under hypoxic and inflammatory conditions and can then form neurotoxic metabolites. When arachidonic acid in the brain undergoes peroxidation and further reactions, it can generate a lipid hydroperoxide and an alkyl radical and ultimately neurotoxic aldehydes (carbonyls) (Yang et al., 2019).

Catecholamine are vulnerable to oxidation because they have a highly reactive quinonoid nucleus. Aberrant oxidation converts catecholamine molecules into quinones that may generate superoxide-free radicals that act as oxidizing agents, damaging neurons (Wang et al., 2021).

### 2.4. Neuroinflammation

Neuroinflammation inducing secondary damage to the brain is heralded by the release of proinflammatory cytokines, chemokines and inflammatory mediators (Ziebell and Morganti-Kossmann, 2010). The resulting inflammatory environment stimulates resident microglia and astrocytes while activated brain endothelial cells cause blood brain barrier (BBB) permeabilization which brings about infiltration of peripheral leukocytes (Acosta et al., 2013; Hernandez-Ontiveros et al., 2013; Liu et al., 2022). Inflammatory mediators are released by these leukocytes, specifically macrophages, neutrophils and lymphocytes, and play a crucial role in neuronal death (Mele et al., 2021). Activated resident microglia further attract immune cells into the injured areas of the brain. Microglial processes represent a first-line defense barrier between healthy and injured areas of the brain (Davalos et al., 2005; Haynes et al., 2006). When activated, microglia release oxidative metabolites such as nitric oxide, ROS, and pro-inflammatory cytokines such as TNF-α, interleukin (IL)-1β, and interferon (IFN)-γ (Lozano et al., 2015). ROS and cytokines then perpetuate the cycle by contributing to brain tissue damage (Kalra et al., 2022). Additionally, chronic complement dysregulation post-injury also plays a critical role in promoting neuroinflammation and neuronal cell death (Dong et al., 2023). Parry and colleagues found that formation of a specific component of the complement system, soluble membrane attack complex C5b-9 (sC5b-9), was elevated in TBI and may be involved in secondary injury and death of neurons (Parry et al., 2020). Toutonji et al. (2021) characterized the expression of 59 complement genes at different time points post-TBI and demonstrated an upregulated gene expression across most complement activation and effector pathways. The study found continued upregulation of C2, C3, and C4 expression up to 2 years following injury. Treatment using the targeted complement inhibitor, CR2-Crry, was also shown to significantly ameliorate TBI-induced transcriptomic changes at all time points after injury.

### 2.5. Cell death

Secondary damage after head trauma ultimately leads to neuronal cell death. Dying neural cells can exhibit either an apoptotic or a necrotic morphology (Raghupathi, 2004; Akamatsu and Hanafy, 2020). Caspases, cysteine proteases activated by proteolytic cleavage, are principal mediators of apoptotic cell death. Excitotoxicity, increased influx of intracellular calcium, as well as the production of free radicals brings about opening of mitochondrial permeability transition pores which leads to cytochrome C release into the cytosol (Bredesen, 2008; Akamatsu and Hanafy, 2020). The cytochrome C forms a complex with apoptotic protease activating factor-1, which then recruits and cleaves inactive procaspase-9 to activate caspase-9, which initiates the apoptotic cascade and activates caspase-3. Caspase-9 and caspase-3 are the key effector enzymes in neuronal apoptosis (Van Opdenbosch and Lamkanfi, 2019). The process that precedes cytochrome C release is thought to involve Bcl-2 family proteins that regulate the permeability of the outer mitochondrial membrane. TBI disturbs the balance between pro-apoptotic BAX, which forms pores in the outer mitochondrial membrane, and anti-apoptotic B cell lymphoma 2 (BCL-2), a pro-survival protein that binds to BAX (Wennersten et al., 2003; Stoica and Faden, 2010; Deng et al., 2020). Numerous natural and artificial caspase inhibitors have been identified and developed with the intention for therapeutic use in reducing neuronal cell death post TBI (Dhani et al., 2021; Feng et al., 2022; Unnisa et al., 2022).

## 3. The pupil and TBI

**FIGURE 2:**
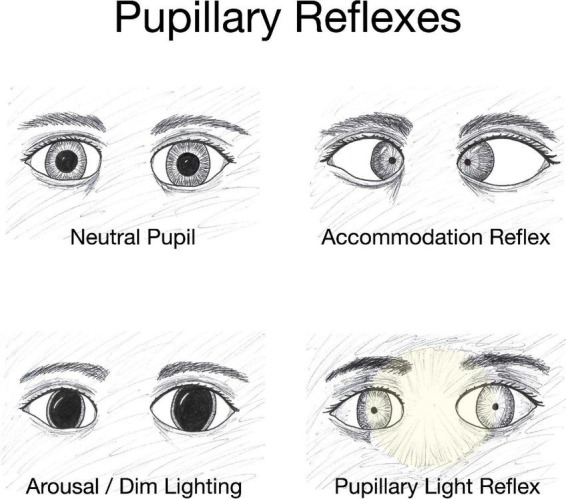
Pupillary reflexes. The diameter of the pupil changes in response to specific conditions such as variations in object distance and level of illumination. The accommodation reflex, an adjustment for near vision, results in pupillary constriction, and inward rotation of the eyes as an object draws nearer. Dim lighting elicits pupillary dilation as does emotional arousal. Increased brightness of lighting causes pupillary constriction and the effect is both direct (affecting the eye exposed to the light) and consensual with constriction of the pupil in the eye opposite to the light-stimulated eye.

Pupils respond to three types of stimuli: they constrict in response to light falling on the retinal photoreceptors *via* the pupillary light reflex (PLR), constrict to bring near objects into focus *via* the accommodation reflex, and dilate in response to increased arousal triggered by strong emotions (Bradley et al., 2008; Mathôt, 2018; Bouffard, 2019; Figure 2). When one eye is exposed to illumination, the pupil of the other eye will constrict in a consensual response. The PLR is involved in both convergence and accommodation, with the parasympathetic system driving constriction through the sphincter pupillae muscles of the iris and the sympathetic system driving dilation through the dilator pupillae muscles of the iris. Dilation is mediated by activity in the locus coeruleus, hypothalamus, and superior colliculus (Mathôt, 2018). It should be noted that both constriction and dilation occur depending on the stimuli and there is a maximum amount the pupil can change. For example, dimming the light falling on the retina causes dilation of the pupils until the pupil diameter reaches maximum opening.

The pupils can be an accessible and reliable indicator of the neural integrity of the visual system (Joshi and Gold, 2020). However, impaired pupillary responses can occur without damage to the neural integrity of the visual system as exemplified by arousal, which can trigger a pupillary response independent of the visual system (Bradley et al., 2008; Tapper et al., 2021). The PLR is more than just a reflex as it is influenced significantly by central brain function (Carrick et al., 2021). Patients who present with TBI commonly have associated pupillary abnormalities (Helmy et al., 2012). Patients with mTBI may present with pupillary responsivity that is significantly delayed, slowed, and reduced compared to the normal population (Ciuffreda et al., 2017; Thiagarajan and Ciuffreda, 2022). The value of quantitative pupillometry as a non-invasive method for assessing TBI is still being explored, but is not established (Master et al., 2020; Traylor et al., 2021).

In cases of severe head trauma, acute pupillary dilation is a neurological emergency. Pupillary dilation is hypothesized to be the consequence of uncal herniation causing mechanical compression of the oculomotor nerve and resulting in brain stem compromise (Ritter et al., 1999; Manley and Larson, 2002). Non-reacting pupils are associated with a poor prognosis (Marmarou et al., 2007). An isolated third cranial nerve palsy can cause anisocoria (uneven pupil size) and can be an ominous sign in the TBI patient. Third nerve palsy can be associated with expanding mass lesions such as extradural and subdural hematomas (Uberti et al., 2021). Anisocoria, especially with exposure to bright light, was shown to correspond to TBI severity in an analysis of prospectively collected registry data on 118 patients with blunt head trauma (Nyancho et al., 2021). An isolated third nerve palsy can also be seen in minor head trauma (Kim and Chang, 2013). The third nerve, also known as the oculomotor nerve, plays an essential role in oculomotor function (Condos et al., 2022). Damage to the third nerve causes efferent impairment of pupillary constriction and thus the pupil will become dilated and poorly responsive to light. As the third nerve has an important role in extraocular movements, in patients with third nerve palsy the eye appears down and turned outward on the ipsilateral side. The third nerve is also important in lid elevation *via* the levator muscle. Significant lid droopiness or ptosis can be a clear sign of third nerve damage (Nagendran et al., 2019).

Many believe that the afferent pupillary defect must be assessed in the TBI patient (Broadway, 2012). In head trauma there is often ocular trauma which can damage the optic nerve of the ipsilateral eye (Qiu et al., 2022). Trauma is not always evident and the decrease in vision may not be profound. The afferent pupillary defect generally does not cause anisocoria and can be a subtle but important finding. Testing for an afferent pupillary deficit involves the swinging flashlight test (Broadway, 2012). The pupil normally constricts to bright light and then redilates when light stimulus is removed. This happens to both pupils as this is a consensual or bilateral phenomenon. In the presence of an afferent pupillary defect, the pupil will paradoxically dilate on the involved side because of a decrease in afferent light input to the optic nerve on that side (Kennedy et al., 2013). Patients with an afferent pupillary defect need a thorough neuro-ophthalmologic evaluation as well as neuroimaging.

In addition to optic nerve damage, local trauma to the eye can damage the efferent loop to pupillary movement and can compromise the sphincter muscles responsible for pupillary constriction. The pupil may no longer be round if damage is asymmetric. Muscle damage can lead to traumatic pupillary dilation (traumatic mydriasis on the ipsilateral side) (Thuma et al., 2022).

Abnormal pupils are often observed in TBI patients with elevated intracranial pressure (Jahns et al., 2019). An early examination is crucial in assessing the TBI with its associated signs and decompressive craniectomy can control the refractory intracranial hypertension. In certain cases of TBI, lasting bilateral mydriasis and an absence of the PLR may be seen. Absence of PLR is generally associated with brainstem damage and a poor prognosis (Chaudhuri et al., 2009; Helmy et al., 2012). There is currently no effective treatment, but surgical craniectomy may be attempted (Athanasiou et al., 2017).

## 4. TBI and contrast sensitivity

Contrast sensitivity, an important characteristic of the visual system, refers to the ability to discriminate between shapes and objects when they do not differ much in brightness or color in comparison to the background and their border may be indistinct (Owsley, 2003; Richman et al., 2013). Eye care professionals commonly use the Snellen eye chart to assess monocular and binocular visual acuity by having the patient identify letters in dark black on a white background on a well-lit screen (Sloan, 1951; Ricci et al., 1998; Tsui and Patel, 2020). However, because these eye charts only measure high-contrast sensitivity, many patients may have excellent visual acuity but present with issues in vision on a day-to-day basis. Contrast sensitivity can be applied to detect aspects of visual quality change beyond acuity and perimetry that relate to driving, facial recognition and various of tasks of daily living (West et al., 2002; Ginsburg, 2003; Swan et al., 2019). Although these tasks involve multiple complex processes, measuring medium and low-contrast sensitivity can be useful, as it may uncover visual defects not seen on a regular eye exam (Boutet et al., 2015). The gold standard for measuring contrast sensitivity involves assessing contrast sensitivity as a function of spatial frequency (contrast sensitivity function), which is time-consuming and complex. Practical clinical measurement may employ pre-printed grating charts, contrast sensitivity letter charts such as the Pelli-Robson or Rabin chart, and computer-based determination of contrast sensitivity thresholds (Elliott et al., 1990; Rabin, 1994; Richman et al., 2015). Paper charts are wall-mounted and require external illumination. They are still widely used, inexpensive and show good reproducibility (Patel et al., 2009).

Traumatic brain injury has been shown to reduce contrast sensitivity (Alnawmasi et al., 2019; Honig et al., 2021). However, it is important to characterize the type of contrast sensitivity impacted; TBI patients have increased sensitivity to first-order motion stimuli, but have decreased sensitivity to contrast-defined and orientation-defined second-order stimuli (Spiegel et al., 2016). First-order stimuli are simply delineated from their backgrounds according to how much the object is illuminated, while second-order stimuli are recognized by the differences in their contrast or texture (Baker and Mareschal, 2001; Lu and Sperling, 2001). By utilizing a motion direction discrimination task, Piponnier et al. (2016) demonstrated that reaction times for second-order stimuli were slower than first-order stimuli in patients who had suffered TBI. Evaluation of contrast sensitivity in TBI patients in addition to testing their visual acuities can lead to more accurate assessment of functional vision. Through more complete visual examination, decreased contrast sensitivity in TBI patients can be detected and managed (Barnett and Singman, 2015). In treating contrast sensitivity deficits, some have advocated utilizing tinted lenses to increase contrast sensitivity, specifically under glare conditions (Lee et al., 2002; Lacherez et al., 2013).

## 5. Flashing lights after TBI

Flashing lights, or photopsia, is a common visual disturbance symptom characterized by seeing brief, flash-like stars in front of the eyes (Virdee and Mollan, 2020). The causes can be numerous and the etiology needs to be investigated. Patients who present with mTBI commonly have associated photopsia (Ciuffreda et al., 2021). In cases of head trauma, seeing stars is generally a transient event resulting from spontaneous firing of neurons in the occipital lobe upon impact and the interpretation of these random electrical impulses as stars by the brain (Moser et al., 2005; Chen et al., 2019).

The crucial clinical question is to ascertain whether the reported phenomena is unilateral or bilateral. Unilateral photopsia (flashing white lights in one eye only) usually originates within the eyeball. Photopsia experienced as flashing lights in both eyes simultaneously is usually central in etiology and represents activity in the brain (Virdee and Mollan, 2020). This is a key clinical distinction and may not be obvious to the patient. Colored lights are almost always of central etiology.

Unilateral photopsia requires a thorough ophthalmologic history and examination. Flashing lights can be accompanied by symptoms of floaters (dark spots in field of vision). This is most commonly caused by a posterior vitreous detachment (Byer, 1994; Verhoekx et al., 2021). The vitreous has attachments to the retina and retraction of these attachments can come about due to forces generated during TBI. This stimulates the retinal cells and is perceived as flashing lights. Tiny particles of debris and sometimes blood can be distributed as a result of this activity and can be perceived as floaters. Usually this vitreous separation is an innocuous event and does not damage the retina. Floaters often become asymptomatic after a few months. However, if there is sufficient traction upon the retina during this separation, a retinal tear can occur. This is a more serious event and can subsequently develop into a retinal detachment where the retina is torn loose from the choroid.

Retinal tears associated with head trauma may present as photopsia (Sornalingam et al., 2018). It is particularly important to assess for retinal tears and retinal detachment when evaluating head trauma because these are emergencies that require prompt intervention (Amos, 1999; Ibrar et al., 2021). Symptoms may be overlooked and attributed to the concussed state (Bedgood et al., 2015). The retina, an extension of the central nervous system (CNS), converts light signals into nerve impulses (Behar-Cohen et al., 2020). Retinal detachment can result in permanent visual loss and thus early examination is critical as tears can be treated to prevent detachment.

Visual snow syndrome (VSS), a rare condition characterized by the appearance of pixelated flickering dots in one plane in front of and throughout a visual field, may be incited by mTBI (Ciuffreda et al., 2021; Hang et al., 2021; Mehta et al., 2021; Werner and Gustafson, 2022). The pathophysiology of VSS is not well-understood, there is no effective treatment and it tends to persist over time (Fraser, 2022).

Bilateral flashing lights is a more complex phenomenon. Visual information is bifurcated at the optic chiasm as it enters the brain, so information is no longer segregated by origin from the left or right eye. This phenomenon going on in the left side of the brain impacts the right visual field and phenomenon going on in the right side of the brain impact the left visual field. Migraine auras are the most common cause of bilateral flashing lights or patterns of lights confined to one hemifield, but, as mentioned earlier, the impact from a TBI event can lead to brief, transient “seeing stars” due to pressure on the occipital lobe.

## 6. Visual and mental fatigue after TBI

### 6.1. Visual fatigue and light sensitivity

Traumatic brain injury can result in visual disturbances and discomfort of which the most common are blurred vision, double vision, light sensitivity, and visual fatigue (Greenwald et al., 2012; Armstrong, 2018). Approximately 50–70% of TBI patients have visual difficulties (Ciuffreda et al., 2007; Greenwald et al., 2012; Berthold-Lindstedt et al., 2017). Photosensitivity is seen in about 50% of mTBI patients and some relief may be given with tinted lenses (Truong et al., 2014). Light sensitivity can be assessed clinically under varying light sources and intensities or by patient self-reporting and validated questionnaires, but there is no established objective test for this somewhat subjective symptom (Truong and Ciuffreda, 2016). The etiology of light sensitivity after TBI is complex and has been postulated to involve damage to intrinsically photosensitive retinal ganglion cells (ipRGC) (Mostafa et al., 2021). These specialized cells are involved in circadian rhythm and other non-image associated functions and can be activated intrinsically *via* the photopigment melanopsin without input from rods or cones. In a mouse model, increases in melanopsin after TBI accompanied light aversion (Honig et al., 2019).

Oculomotor dysfunction is very common after TBI, with estimates of frequency of occurrence between 60 and 85% (Ciuffreda et al., 2007). Oculomotor dysfunction, attributed to damage to efferent pathways, is due to effects on cranial nerves, nerves controlling eye movements and vestibular pathology (Thiagarajan et al., 2014). When control of the position and movement of the eyes is impaired, there can be a delay in shifting focus between close and far fields, trouble with keeping focus on a close object, vergence-related abnormalities, and difficulties with tracking moving objects (Poltavski and Biberdorf, 2014; Kontos et al., 2017; Reneker et al., 2018). The consequences of oculomotor dysfunction may cause a person to skip lines when reading, perceive movement of print when reading, and experience eye strain (Capó-Aponte et al., 2017; Kapoor and Ciuffreda, 2002). Individuals with mTBI show complications in functional task abilities when engaging in reading comprehension. In reading tasks, those with mTBI exhibit more fixation time when reading words with lower frequencies. Overall, mTBI patients may display weaker functioning in reading in terms of accuracy, time of fixation, and a lower number of blinks (Ratiu et al., 2022). Sustained reading becomes very difficult and eyestrain is common. Convergence insufficiency with hampered ability to focus on a near target makes reading an arduous task. The need for ocular muscles to compensate for injury-induced diminished responsiveness leads to eyestrain and eye muscle fatigue. Headache may ensue (Ciuffreda et al., 2007). Patients with vergence problems may attain some relief with specially designed eyeglasses containing prism lenses and may be prescribed specific eye exercises (Aletaha et al., 2018).

### 6.2. Mental fatigue

In addition to visual fatigue, mental fatigue and feeling tired with low mental energy are common after TBI and can interfere with everyday living and fulfilling job responsibilities (Cantor et al., 2008; Johansson et al., 2009). It has been postulated that mental fatigue occurs because the injured brain is not working efficiently and has to expend extra energy to perform tasks that previously did not deplete reserves (Kohl et al., 2009). The brain becomes overloaded at low threshold and recovery is slowed compared to pre-trauma. When performing working memory tasks, the traumatized brain exhibits increased activity progressively over time in bilateral dorsolateral prefrontal cortex and the inferior parietal brain regions as a compensatory mechanism to meet demand and that this results in fatigue (Dettwiler et al., 2014; Sun et al., 2014). Trauma-induced changes in regional cerebral blood flow (rCBF) may help to explain fatigue symptoms. Magnetic resonance imaging studies show that self-assessment of perceived fatigue during a psychomotor vigilance task correlates to changes in rCBF (Möller et al., 2017). The rCBF differed in mTBI patients compared to healthy controls during the task in multiple brain regions including the left thalamus and superior frontal gyri, right precuneus and insula, and left/right medial frontal gyri and anterior cingulate cortex. During the task, the mTBI patients exhibited fatigue as slowing of performance over time. A correlation was found between self-perceived fatigue after a psychomotor vigilance task and high rCBF in the left medial frontal and anterior cingulate gyri. In a study by Ramage et al. (2019), pattern of functional connectivity, reflecting communication between brain regions, differed between control and mTBI subjects performing a behavioral task with heightened connectivity across all effort levels for the TBI subjects. This may reflect inefficiency of the activity of the neural network that leads to fatigue.

### 6.3. The intersection of mental fatigue and visual fatigue

Although the causes of the experience of mental exhaustion are not known, a correlation has been found between number of visual symptoms and self-reported feelings of mental fatigue (Berthold Lindstedt et al., 2019). The connection between visual processing and mental fatigue after TBI crystallizes in the work of Prak et al. (2021) in which a correlation was seen between self-reported fatigue and increased activation of the bilateral visual cortices in areas associated with motion perception, attention, and oculomotor pursuit.

Insomnia is a common symptom after TBI that may contribute to both eye fatigue and mental fatigue (Jain et al., 2014; Ouellet et al., 2015). Not surprisingly, there is a positive association between sleep disruption and fatigue in TBI patients (Englander et al., 2010; Cantor et al., 2012). The pattern of sleep disruption in TBI is heterogeneous and difficult to characterize (Raikes et al., 2019). In order to explore sleep characteristics of mTBI patients, Arbour et al. (2015) evaluated 34 mTBI subjects and 29 age-matched controls using polysomnographic recording at a sleep laboratory and found little difference in non-rapid eye movement (NREM) sleep patterns. They did find the mTBI group had increased beta power in the occipital derivation compared to the control group in all sleep cycles, which may result from injury to the brain as well as anxiety and pain. The eyes may be affected by insufficient sleep, leading to ocular discomfort, dry eyes, and itching (Kawashima et al., 2016; Li et al., 2018). Sleep disorders may make reading, driving, and viewing computer screens more difficult and uncomfortable (Engle-Friedman et al., 2018; Gibbings et al., 2022).

Chronic post-traumatic headaches are a reoccurring issue for TBI patients that can augment both visual and mental fatigue (Faux and Sheedy, 2008; Voormolen et al., 2019; Ashina et al., 2020). The headaches may resemble a tension headache or have migraine-like qualities and may be accompanied by nausea, vomiting, and photophobia (Ashina et al., 2019). Nearly every type of headache listed by the International Headache Society can be linked to vision disorders after TBI (Dwyer, 2018; Quaid and Singman, 2022). Again, reading or looking at a computer screen becomes more challenging when headache is present. Eye fatigue and mental fatigue overlap in their ability to compromise quality of life after TBI, and both are exacerbated by disrupted sleep and headaches.

## 7. TBI and falls in older persons

For older persons, the rate of fall-induced TBIs is almost twice that of younger persons (Haring et al., 2015). Ground-level falls are the most common cause of injury in the geriatric population and the prevalence of fall-related TBI is steadily increasing (Harvey and Close, 2012; Korhonen et al., 2013; McGuire et al., 2017; Miyoshi et al., 2020). In the United States in 2013, TBI accounted for over 434,000 (2,232.2 per 100,000 population) emergency department visits, hospitalizations, and deaths in persons aged 75 and older (Thompson et al., 2006). TBI in older adults is a major cause of morbidity and mortality and a significant health and socioeconomic problem (Bailey et al., 2022). Over 60% of TBI cases in older individuals are due to unintentional falls, which become more likely as people age due to muscle weakness, balance issues, deteriorating vision, and effects of some widely used medications (Gale et al., 2016).

Balance problems are another major issue faced by our aging population. In fact, roughly one in five elderly persons experience problems related to dizziness or balance annually (Lin and Bhattacharyya, 2012). Certain medications may increase the likelihood of falls among the elderly (Gillespie et al., 2012; Hopewell et al., 2018). Hart et al. (2022) found that the dose of CNS-active medications, which include antidepressants, opioids, and benzodiazepines, was not reduced appreciably following a fall-related injury in older adults. This suggests that adjustment in CNS-active medications is not being utilized adequately to modify fall risk. Furthermore, Evans et al. (2011) conducted a study to gain a better understanding of the impact of multiple medication utilization by trauma patients 45 years and older. The study demonstrated that over 40% of the trauma patients were receiving five medications or more at the time of injury. As such, the patients were at a greater risk for complications, lower functional outcomes, and longer hospitalizations. Consequently, decreasing concurrent medication use may be beneficial for trauma patients and should be investigated further (Evans et al., 2011).

Traumatic brain injury in geriatric patients can have a compounding effect on vision in persons who may already have diabetic retinopathy, glaucoma, macular degeneration, cataracts, and other conditions (Reed-Jones et al., 2013; Ehrlich et al., 2019; Teo et al., 2021). Depending on location and severity, TBI can lead to damage to the optic nerve, optic tract, and occipital lobe. These injuries can manifest as blurred vision, double vision, and/or decreased peripheral vision (Sen, 2017). In order to avoid obstacles, older persons need to safely navigate their environment and, when visual input is insufficient, may require behavioral or assistive technology (Patla, 1997; Marigold and Patla, 2008; Slade et al., 2021). Visual impairments in the elderly are a major contributing factor in falls (Coleman et al., 2007; Freeman et al., 2007).

Falls may, of course, result in hospitalization without impacting the head or causing TBI, but falls are the most common cause of TBI in older adults (Fu et al., 2017; Hofmann et al., 2021). Intracranial hemorrhage can occur as a consequence of TBI and is one of the more serious potential complications associated with falls (de Wit et al., 2020a). Falling on level-ground is the most common cause of intracranial bleeding. de Wit et al. (2020b) found that 1 in 20 seniors who presented to the emergency department after a ground-level fall are diagnosed with intracranial bleeding. As such, patients taking anticoagulants are at an increased risk of intracranial hemorrhage and should be monitored appropriately. The major increase in TBI-related hospitalizations is driven by falls in the elderly and these falls are leading to a significant increase in diagnosed intracranial hemorrhages (Harvey and Close, 2012; Nederpelt et al., 2022).

Traumatic brain injury can lead to the development of cerebral microbleeds (CMB), small hemosiderin deposits that come from bleeding of injured small arteries, cerebral arterioles, or capillaries (Lee et al., 2018). CMB are associated with gait and balance issues as well as progressive cognitive decline (Altmann-Schneider et al., 2011; Yates et al., 2014; Yakushiji, 2015; Ungvari et al., 2017; Irimia et al., 2018). CMB are associated with slower processing speed, defective attention and executive function, and they have been linked to promoting major depressive episodes (Niogi et al., 2008; Wang et al., 2014; Akoudad et al., 2016). Aging has been shown to exacerbate microvascular fragility and promote the formation of CMB (Toth et al., 2021). The prevalence of CMB increases with advancing age and approaches 20% by age 65 (Vernooij et al., 2008; Altmann-Schneider et al., 2011; Ungvari et al., 2017; Irimia et al., 2018). As TBI can affect the elderly population disproportionately, it is important to monitor older persons in order to prevent any adverse effects resulting from CMB.

Traumatic brain injury in older persons is associated with poorer functional outcomes, higher risk of mobility restriction, permanent disability, and loss of independence (LeBlanc et al., 2006; Gardner et al., 2018; Gavrila Laic et al., 2021; Tyler et al., 2022). While the reasons for poorer outcomes are not fully known, the high incidence of comorbidities that accompany aging may contribute (Winter et al., 2022). Another factor may be the higher proportion of older persons using warfarin or other anticoagulants that increase risk of brain bleed with head trauma, leading to poorer outcomes (Zeeshan et al., 2018; Giner et al., 2022). Mortality is also extremely common following a TBI amongst this aging population (Susman et al., 2002; Sylliaas et al., 2009). Prevention of falls in older persons may involve multiple initiatives designed to minimize risk and improve stability, including balance and strength training, securing appropriate footwear, minimizing polypharmacy, appropriate vision aids, and environmental modifications (Moncada and Mire, 2017).

## 8. TBI and seizures

Patients who present with moderate to severe TBI often have associated seizures (Fordington and Manford, 2020). Seizures that occur immediately (within 24 h of trauma) are not considered “epileptic” and are attributed to the impact itself (Agrawal et al., 2006). Epilepsy developing after an acute brain insult is referred to as post-traumatic epilepsy (PTE) (Verellen and Cavazos, 2010; Anwer et al., 2021). In evaluating any patient presenting with a seizure of unclear etiology, history of head injury is crucial information. Seizure activity can worsen the consequences of TBI by depriving the brain of oxygen and causing release of inflammatory mediators. The seizure itself can act like a second brain injury (Mazzeo and Gupta, 2018). In mTBI, seizures are less common, but if a seizure occurs in the emergency room, or shortly after discharge, this can be a warning sign that important findings of significant intracranial injury may have been missed (Vaniyapong et al., 2020).

Traumatic brain injury is a key cause of PTE, a condition in which recurrent, unprovoked chronic seizures occur 2 weeks or more after the TBI event (Campbell et al., 2014). PTE is a common form of acquired epilepsy representing 5% of all cases of epilepsy (Fordington and Manford, 2020). It is estimated that between 3 and 5% of moderate TBI cases and between 25 and 50% of severe TBI cases go on to have PTE (Agrawal et al., 2006).

Epileptogenesis, the process through which changes occurring in the brain lead to seizures, is hypothesized to begin at the moment of the trauma itself, even though the latent period before epilepsy develops can last weeks, months or even years after the inciting injury (Benardo, 2003). Seizures tend to persist over time (Annegers et al., 1998; Steinmetz et al., 2013). The seizures originate in perilesional cortical and mesiotemporal regions (Englander et al., 2014; Perucca et al., 2019; Tubi et al., 2019). Although the pathways that lead from brain injury to seizure are not completely understood at the molecular level, PTE is thought to result from neuroinflammation, oxidative stress and neuronal loss (Li L. et al., 2021; Reddy et al., 2022). Predictors for likelihood of developing PTE include the presence of intracranial bleeding and more severe TBI (Englander et al., 2003; Pease et al., 2022).

There is currently no treatment to prevent onset of PTE, but it is imperative to evaluate patients for seizure activity immediately post-TBI as antiepileptic drugs can be given to manage associated symptoms (Englander et al., 2014; Reddy et al., 2022). An electroencephalogram (EEG) should be used when evaluating TBI patients because up to 25% of these patients may have sub-clinical seizure activity on EEG (Ronne-Engstrom and Winkler, 2006; Chen and Koubeissi, 2019). While drug treatment is generally effective in seizure control, some PTE patients are medication-resistant and difficult to manage (Gupta et al., 2014; Gugger et al., 2022).

Traumatic brain injury can also lead to psychogenic non-epileptic seizures (PNES) (Barry et al., 1998; Hingray et al., 2016). PNES resemble epileptic seizures in their symptoms, but are not caused by abnormal electrical discharges in the brain (Auxéméry et al., 2011). Rather, they are a type of non-epileptic seizure linked to underlying psychosocial stressors (LaFrance and Devinsky, 2002; Popkirov et al., 2018). PNES can co-occur in patients with epilepsy and can be a diagnostic challenge (El-Naggar et al., 2017). The misdiagnosis of PNES leads to ineffective treatments and poor patient outcomes (Barry et al., 1998; Gorenflo et al., 2022). Carefully working through the differential diagnosis with consideration of PNES after TBI will lead to appropriate treatment, which, for PNES generally consists of psychotherapy and psychopharmacological approaches (Carlson and Nicholson, 2017; Lopez and LaFrance, 2022).

## 9. Brain repair after TBI

As we described in Section “2. Cellular response to traumatic brain injury,” following TBI there is cell death and inflammation and the brain reacts in numerous ways that may be harmful or helpful. Many cell types participate in the process of healing and resolution. Support cells such as astroglia and microglia play an important role in determining recovery outcomes. This section discusses the post-TBI repair process at the cellular level.

### 9.1. Reactive astrogliosis

Astrocytes, the most abundant glial cell type within the CNS, are essential in maintaining CNS physiological homeostasis. This cell type maintains the integrity of the BBB, supports neuronal function, scavenges free radicals, and regulates extracellular glutamate (Cheng et al., 2019). In response to TBI and other CNS insults, astrocytes undergo a series of morphological and functional adaptations referred to as reactive astrogliosis. Astrogliosis is often referred to as a scar-forming process that occurs around a lesion marked by cell hypertrophy and proliferation and changes in gene expression (Cheng et al., 2019; Sofroniew, 2020; Li J. et al., 2021; Ayazi et al., 2022). Reactive astrocytes proliferate rapidly, densely packing and enclosing the injured area. They secrete inflammatory mediators and neurotrophic factors, and increase expression of intermediate filaments such as glial fibrillary acidic protein (GFAP) and vimentin (Herrmann et al., 2008; Karve et al., 2016; Gill et al., 2018; Hemati-Gourabi et al., 2022). Although this process can perpetuate detrimental CNS injuries through neuroinflammation and ROS generation, reactive astrogliosis can limit damage by forming a physical barrier that protects surrounding healthy tissue while also eliciting reparative effects through promotion of neurogenesis and synaptogenesis (Sheng et al., 2013; Liddelow and Barres, 2017; Adams and Gallo, 2018; Moulson et al., 2021). Thus, depending on the surrounding environment, the state of astrogliosis can manifest as either a neuroprotective or neurotoxic phenotype (Liddelow et al., 2017).

Some potential mechanisms of astrocyte-induced neuroprotection in TBI have been proposed (Song et al., 2002; Bylicky et al., 2018). Astrocytes produce the neurotrophic and mitogenic calcium binding protein S100β, which enhances neurogenesis within the hippocampus. Serum level of S100β is a well-established biomarker and predictor of CT abnormalities for early mTBI (Kahouadji et al., 2020). In male rats, hippocampal infusion of S100β following TBI improved cognitive functional recovery (Hayakata et al., 2004; Kleindienst et al., 2005). These effects are mediated by the facilitation of neuronal differentiation, proliferation, and survival of hippocampal progenitor cells (Hinkle et al., 1997; Kleindienst et al., 2013; Baecker et al., 2020; Zhou et al., 2020). Heme oxygenase induced by astrocytes after TBI catalyzes heme to carbon monoxide (CO), ferrous iron, and biliverdin. Choi (2018) demonstrated that low concentrations of CO promote neurogenesis, synaptic plasticity, and angiogenesis (Jung et al., 2020).

Studies are being done to find ways to manipulate astrocytes to control their reactivity and shift emphasis toward healing. In a mouse model of TBI, Wu et al. (2023) found that brain derived neurotrophic factor (BDNF) secretion by an activated astrocyte network could form a gradient to guide neuroblasts to the site of injury where they could potentially differentiate and facilitate repair.

Mature astrocytes can regress to an immature phenotype and show stem cell characteristics which could indicate a possible role in neuronal regeneration (Seri et al., 2001; Matsubara et al., 2021). Further, exosomes derived from healthy cultured primary rat astrocytes can protect against cognitive dysfunction, oxidative stress, and apoptosis in a rat model, pointing toward a possible therapeutic approach to attenuating TBI injury (Zhang et al., 2021).

### 9.2. Microglial activation

Microglial cells are innate immune cells of the CNS that promote repair through diverse mechanisms (Lyu et al., 2021). A dichotomous classification has been adopted to characterize microglia polarization into either a classically activated (M1) or an alternatively activated (M2) phenotype (Orihuela et al., 2016). M1 microglia lead to neuronal damage and brain dysfunction through pro-inflammatory factors, such as IL-1β, IL-6, and TNF-α. In contrast, M2 microglia are reparative in nature and reduce toxic cellular debris through phagocytosis, release neurotrophic factors, and resolve cerebral inflammation (Hu et al., 2015; Chai M. et al., 2022). Anti-inflammatory M2 microglia secrete neuroprotective cytokines, chemokines, and neurotrophic factors that contribute to BBB protection, remyelination, neurogenesis, angiogenesis, and axon regeneration (Yang et al., 2015; Ronaldson and Davis, 2020; Lyu et al., 2021). Microglia play a role in rapid closure of the BBB *via* chemotaxis of microglial processes after brain injury, a process mediated by the purinergic receptor P2YG protein–coupled 12 (P2RY12) (Lou et al., 2016; Császár et al., 2022). Choi et al. (2017) demonstrated M2 microglia-conditioned media induced by IL-4 increased the proliferation and differentiation of neural stem progenitor cells (NSPCs) in the ipsilateral subventricular zone of *ex vivo* ischemic brain sections. M2 microglia promote axonal regeneration through the secretion of protective molecules, such as arginase 1 (an enzyme that contributes to extracellular matrix deposition in wound healing) and BDNF (Louveau et al., 2015). Similarly, the anti- inflammatory phenotype may improve angiogenesis after brain injury through production of neuroprotective vascular endothelial growth factor (VEGF) and IL-8 (Medina et al., 2011). Unfortunately, anti-inflammatory treatments, including steroids, have not shown efficacy in improving TBI outcomes (Begemann et al., 2020).

### 9.3. Neuroinflammation

Neuroinflammation can often result in the uncontrolled release of toxic cytokines, proteases, glutamate, and free radicals. These effects are deleterious in nature to the injured CNS (Wee Yong, 2010; Simon et al., 2017). However neuroinflammation is not always synonymous with poor CNS outcomes (Bollaerts et al., 2017). T lymphocytes are a subset of inflammatory cells that have been found to facilitate axonal regeneration (Hauben et al., 2000). Ishii et al. (2012) demonstrated that the adoptive transfer of CD4+ T helper Th1, but not Th2 or Th17 cells, 4 days after traumatic spinal cord injury was associated with regrowth of the corticospinal tract and serotonergic fibers promoting locomotor and tactile recovery. Leukocytes and microglia are notable producers of neurotrophic factors, including oncomodulin, osteopontin, platelet-derived growth factor (PDGF), epidermal growth factor (EGF), fibroblast growth factor-2 (FGF-2), ciliary neurotrophic factor (CNTF), activin-A, glial-derived growth factor (GDNF), endothelin-2, insulin-like growth factor-1 (IGF-1), BDNF and neurotrophin-3 (Sousa-Victor et al., 2018). Many of these neurotrophic factors have been shown to be beneficial for the proliferation and differentiation of oligodendrocyte progenitor cells and for neurogenesis (Woodruff et al., 2004; Yuen et al., 2013; Yong et al., 2019).

While care of TBI patients is largely supportive, there is a growing understanding of the reparative process initiated within the CNS after injury. The ability of the brain to recover from injury is limited, but as new experimental data is acquired, targets for intervention to facilitate repair and regeneration may be identified and explored (Jin et al., 2022; Prabhakar et al., 2022).

### 9.4. Stem cells

The use of stem cells for neural regeneration and restoration after TBI is an exciting cutting-edge area of exploration. The neurogenic regenerative capacity of endogenous neural progenitor cells has been reported in brain injury models in animal and in human studies (Macas et al., 2006; Sun, 2016). In the adult brain, endogenous neural stem cells are primarily localized to the subventricular zone of the lateral ventricles and the subgranular zone of the hippocampal dentate gyrus (Gage et al., 1998; Adugna et al., 2022). Recent studies targeting approaches to enhance the proliferation of these endogenous neural stem cells after TBI have demonstrated promising results. Chai Y. et al. (2022) showed in a rat brain injury model that transplanting an aligned fibrin hydrogel scaffold into the injury site promoted effective migration, differentiation, and maturation of endogenous neural stem cells, resulting in neurological functional recovery. This highly biomimetic scaffold mimics the parallel oriented structure of radial glia in the embryonic brain, a cell type that guides the directed migration of neurons in response to brain injury.

Transplantation of exogenous stem cells is also being investigated as a way to overcome the limited regenerative capabilities of the brain after TBI (Dekmak et al., 2018). Exogenous stem cell transplantation has been shown to accelerate immature neuronal development and increase endogenous cellular proliferation in damaged brain regions (Tajiri et al., 2013; Ngwenya et al., 2018; Adugna et al., 2022). Mesenchymal stromal cells (MSC) are multipotent stem cells with self-renewal and multi-differentiation abilities. Recent studies have demonstrated that exogenous MSC have the potential to treat TBI *via* their anti-inflammatory and antiapoptotic properties (Ferreira et al., 2018). In a rat model, MSC can also form a biobridge facilitating migration of endogenous neurogenic cells to the injured site (Tajiri et al., 2013). In the context of TBI, the secretome of MSC has the capacity to enhance endogenous neurogenesis (Liu et al., 2020; Badner and Cummings, 2022). Porcine and rodent models show that extracellular vesicles released by MSC can promote endogenous angiogenesis and neurogenesis, reduce inflammation, and facilitate cognitive and sensorimotor recovery after TBI (Abedi et al., 2022; Bambakidis et al., 2022). This highlights the significance of the trophic support these cells provide during exogenous application (Spees et al., 2016; Liu et al., 2020). Animal models have demonstrated reduced pro-inflammatory cytokine expression levels of IL-6, IL-1α, and IFN- γ after MSC transplantation *via* intraventricular infusion (Huang et al., 2019). Administration of autologous bone marrow MSCs (BM–MSCs) to patients during the subacute phase of TBI also resulted in improved neurological function in 40% of patients (Tian et al., 2013).

The therapeutic application of neural stem cell treatment, whether *via* manipulation of endogenous neural stem cells or implantation of exogenous neural stem cells, has notable potential to foster functional recovery in those manifesting TBI-related disability. However, further studies are needed to evaluate the safety and efficacy of stem cell transplantation in TBI in humans (Schepici et al., 2020).

## 10. Conclusion

Traumatic brain injury affects multiple aspects of neurologic and cognitive function, while also causing substantial pain and discomfort. The effects may be long-lasting and poorly responsive to treatment attempts. The intimate link between brain and eye leads to many manifestations of TBI that disturb vision, perception, and ability to perform essential everyday tasks such as reading, typing, driving, and navigating within the environment. Headaches, fatigue and eyestrain compound these problems. Although healing after TBI is difficult, intensive rehabilitation and interdisciplinary treatment including cognitive-behavioral therapy can improve overall functional outcomes (Vanderploeg et al., 2019; Alashram et al., 2022). Rescue or reprogramming of brain cells may be possible in the future, but the leap from animal models to humans is a large one and, at this time, there is no intervention with proven effectiveness to offer persons with TBI (Xu et al., 2022). Enhancing neural regeneration using stem cells is promising and is an area of active investigation.

## Author contributions

AR and SR conceptualized the manuscript. AR, AZ, BJ, and DR wrote the initial manuscript and figures and further developed the manuscript. DP and AP provided meaningful edits in the draft phases of the review and contributed meaningful feedback in the writing process. All authors contributed to the article and approved the submitted version.
